# Enhancing papaya resistance to ringspot virus through CRISPR/Cas9-mediated gene editing of e*IF4E*

**DOI:** 10.3389/fpls.2026.1799408

**Published:** 2026-05-19

**Authors:** Ngoc Thu Le, Trang Huyen Thi Hoang, Huyen Thi Tran, Thao Phuong Bui, Quyen Phan, Ha Hoang Chu, Yue Fei, Robert O. Mason, Attila Molnar, Phat Tien Do

**Affiliations:** 1 Institute of Biology, Vietnam Academy of Science and Technology, Hanoi, Vietnam; 2 University of Science and Technology of Hanoi, Vietnam Academy of Science and Technology, Hanoi, Vietnam; 3 Graduate University of Science and Technology, Vietnam Academy of Science and Technology, Hanoi, Vietnam; 4 Institute of Molecular Plant Sciences, University of Edinburgh, Edinburgh, United Kingdom; 5 National Key Laboratory for Tea Plant Germplasm Innovation and Resource Utilization, Anhui Agricultural University, Hefei, China

**Keywords:** CRISPR/Cas9, eukaryotic translation initiation factor 4E(eIF4E), papaya, papaya ringspot virus (PRSV), resistance

## Abstract

Papaya is a tropical fruit crop with high nutritional and medicinal properties. Its production worldwide is significantly hindered by papaya ringspot disease, caused by a potyvirus known as papaya ringspot virus (PRSV). While the eukaryotic translation initiation factor 4E gene (*eIF4E*) and its paralogue *eIF(iso)4E* have been linked to potyvirus resistance in various model and crop species, their role in PRSV susceptibility has not been systematically investigated in papaya. We employed the CRISPR/Cas9 gene editing technology to create gene editing reagents that induce two independent double-strand DNA breaks at the *eIF4E* and *eIF(iso)4E* target genes. We then regenerated stable transgenic plants by *Agrobacterium*-mediated transformation using somatic embryos. Heteroduplex and Sanger sequencing analyses revealed chimeric and heterozygous mutations in *eIF4E* and *eIF(iso)4E* genes in the regenerated transgenic papaya T0 lines, respectively. Notably, only *eIF4E*-edited plants demonstrated resistance to PRSV across all time points tested. Both DAS-ELISA and qPCR analyses confirmed undetectable viral accumulation in these mutant lines. In contrast, PRSV accumulation was observed in the systemic leaves of both wild-type (WT) and *eIF(iso)4E*-edited lines, which developed characteristic symptoms over time. These results identify eIF4E as a critical host factor for PRSV and show that targeted mutations can confer robust resistance. These highly resistant lines will serve as foundational germplasm for further selection and breeding. Despite the limitations of using chimeric T0 mutant lines, this study establishes a framework for developing improved local papaya cultivars through advanced gene-editing technologies.

## Introduction

Papaya (*Carica papaya* L.), belonging to the family Caricaceae, is a tropical fruit crop known for its high nutritional and medicinal values (Hoang et al., 2022). Originally from Central America and Mexico, it is now cultivated in most tropical and sub-tropical countries, especially in Africa and Asia (Kaur et al., 2018). The fruit is low in calories but rich in antioxidants such as vitamin A, C, and E (Minuye, 2024). Other parts of the papaya plant, including the leaves, roots, flowers and seeds also provide medicinal benefits for gut health and immune function (Ali et al., 2012).

In 2023, papaya was one of the most traded commodities globally, with a total trade volume of $363M (https://oec.world/en/profile/hs/papayas-fresh). However, the global production of papayas faces significant challenges due to papaya ringspot disease, caused by the papaya ringspot virus (PRSV) (Ansari et al., 2023). PRSV is a single-stranded RNA virus from the Potyviridae family, which is mainly transmitted by aphids in a non-persistent manner. Infected papaya plants exhibit severe symptoms, including stunted growth, chlorosis, leaf distortion and curling, as well as fruits showing darkened rings and reduced nutritional value (Jia et al., 2017). These infections can lead to yield losses as high as 100%. Traditional methods and breeding approaches to control PRSV disease are often less effective and time-consuming (Jyotika et al., 2024).

The first PRSV-resistant transgenic papaya plants were developed in the early 1990s in the USA. Varieties such as Rainbow and SunUp, which are tolerant to PRSV were among the first to be commercialized (Wu et al., 2018). Research in other countries like Thailand, Malaysia, Philippines, and China has also led to successes in developing PRSV-resistant transgenic papayas using coat protein-mediated and RNA silencing-based mechanisms (Tennant et al., 2001; Wu et al., 2018; Ye and Li, 2010; Jia et al., 2017). Despite these advances, there are significant limitations, including inconsistent resistance, off-target effects, and public concerns about genetically modified (GM) crops (Tabein et al., 2020; Taliansky et al., 2021; Akbar et al., 2022).

Recently, the CRISPR/Cas technology has been successfully applied to enhance viral resistance in plants (Pyott et al., 2020; Jeyaraj et al., 2024). CRISPR reagents can be programmed to target and cleave viral DNA or RNA, or edit/mutate host factors required for viral infection and replication, such as the eukaryotic translation initiation factor 4E (eIF4E) or its paralogue eIF(iso)4E (Varanda et al., 2021). Unlike RNAi-mediated silencing, the CRISPR/Cas9 system induces targeted double-strand breaks in the DNA, enabling complete gene knockouts that confer stable, heritable resistance. Furthermore, the development of transgene-free mutant lines, achieved through genetic segregation in subsequent generations, is set to circumvent the regulatory and public perception challenges typically associated with the commercialization of traditional genetically modified (GM) crops. Knockout of *eIF4E* and *eIF(iso)4E* genes has been proven to confer virus resistance in both dicots such as Arabidopsis, cucumber, melon, and tomato (Pyott et al., 2016; Chandrasekaran et al., 2016; Pechar et al., 2022; Kumar et al., 2022) and monocot plants such as barley, wheat (Hoffie et al., 2021; Kan et al., 2023). However, to date, the contribution of *eIF4E* and *eIF(iso)4E* genes to PRSV susceptibility in papaya has not yet been systematically investigated. In this report, we applied CRISPR/Cas9 to edit the papaya *eIF4E* and *eIF(iso)4E* genes separately. We obtained targeted mutations in the T0 generation and demonstrated that the *eIF4E* gene is critical and essential for resistance against PRSV infection in papaya.

## Materials and methods

### Bacterial strains, plant material and growth conditions

Immature seeds of the local papaya cultivar Linhan were supplied by the Fruit and Vegetable Research Institute (Ha Noi, Vietnam). *Agrobacterium tumefaciens* strain AGL1 used for papaya transformation was provided by the Laboratory of Plant Cell Biotechnology, Institute of Biology, Vietnam Academy of Science and Technology. WT and transgenic papaya plants were grown in potting mix (1/3 vermiculite + 2/3 peat moss) at 25 ± 2°C with 80% relative humidity under 16-h light/8-h dark photoperiod in growth chambers.

### Target selection, single guide RNA design and CRISPR/Cas9 vector construction

**Figure 1:**
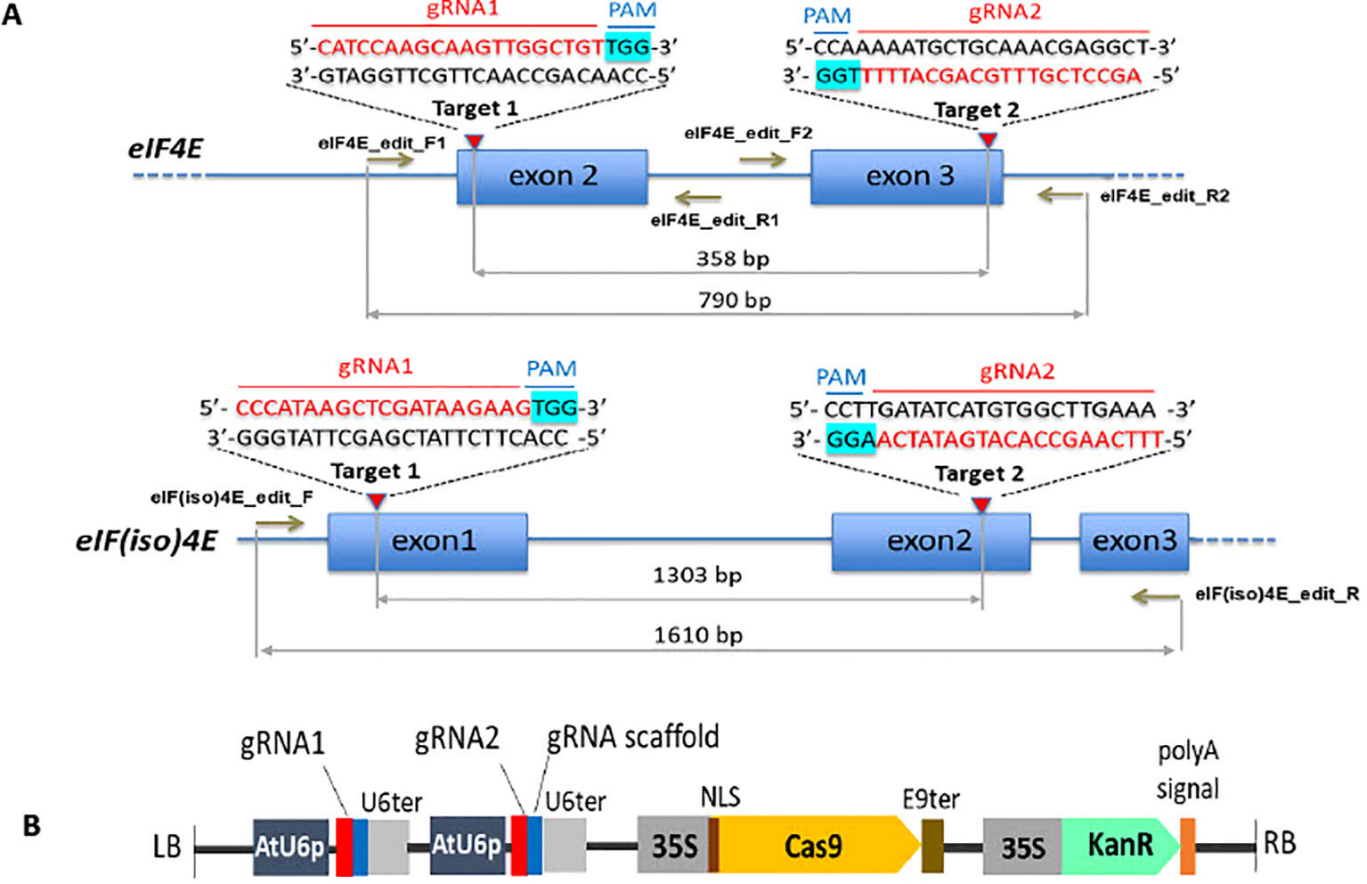
Schematic of the CRISPR/Cas9 gRNA target locations and the CRISPR/Cas9 constructs for *eIF4E* and *eIF(iso)4E* gene editing in papaya. **(A)** Position and sequence of the CRISPR/Cas9 gRNA target sites in the papaya *eIF4E* and *eIF(iso)4E* genes. The gRNA sequences and the protospacer adjacent motifs (PAM) are highlighted in red and magenta, respectively. The arrows indicate the position of PCR primers used for genotyping and sequencing. **(B)** Composition of the CRISPR/Cas9 constructs pKSE401-eIF4E and pKSE401-eIF(iso)4E. The expression of gRNAs is controlled by the Arabidopsis U6 promoter (AtU6p) and the Arabidopsis U6 terminator (U6ter); Cas9: Maize-codon-optimized *Cas9* gene is driven by the 35S promoter (Cauliflower mosaic virus promoter) and terminated by the Pea RuBisCO small subunit E9 terminator (E9ter); Nuclear localization signal (NLS); Kanamycin resistance gene (KanR); T-DNA left and right border sequences (LB, RB).

The partial genomic sequences of *eIF4E* and *eIF(iso)4E* genes from the local papaya cultivar Linhan were cloned and sequenced using the *Carica papaya eIF4E* gene (GenBank accessions HQ013137.1, FJ644949.1) and *Carica papaya eIF(iso)4E* gene (Phytozome genome ID: 113, evm.TU.supercontig_44.11) as references. To induce targeted mutations, two independent Cas9 target sites, named gRNA1 and gRNA2, were identified by CCTop (https://cctop.cos.uni-heidelberg.de:8043/) (Stemmer et al., 2015) for each gene (**Figure 1A**). Two dual-gRNA-CRISPR/Cas9 constructs were then generated using the pKSE401 vector (Addgene, #62202) (Xing et al., 2014) following two main steps: (1) two fragments of BsaI-gRNA1-gRNA scaffold-U6 terminator-BsaI and BsaI-U6 promoter-gRNA2-BsaI were amplified from the pKSE401 template using the listed primers (**Supplementary Table 1**); (2) Next, the corresponding PCR fragments were parallelly assembled into pKSE401 at the *Bsa*I sites to yield the final vector pKSE401-eIF4E or pKSE401-eIF(iso)4E, respectively. These CRISPR/Cas constructs were subsequently introduced into *A. tumefaciens* strain AGL1 for stable papaya transformation.

### *Agrobacterium*-mediated papaya transformation and transgene confirmation

Papaya transformation was performed according to Ying et al., 1999, with some modifications (**Supplementary Table 2**, **Supplementary Figure 1**). Briefly, immature papaya fruits at 80–90 days after pollination were washed under running water with soap (2–3 times). Then, 70% ethanol was used to clean the surface of the papaya fruits, and sterilized knives were used to collect the seeds. Next, papaya immature zygotic embryos were excised from seeds and placed onto the callus induction medium. Explants were sub-cultured every 4 weeks, and somatic embryos were collected for *Agrobacterium*-mediated transformation at 14–16 weeks. Notably, the white, spongy somatic embryos were selected and transferred into the liquid initiation medium and subsequently incubated for 2 days at 150 rpm, 28°C. Then, the embryos were immersed into an *Agrobacterium* suspension at OD_600nm_ = 0.3 for 30 min. After co-cultivation, the embryos were moved onto sterile filter papers to remove excess bacterial suspension and then transferred to the cultivation medium for 3 days at 25°C in the dark. Subsequently, the explants were washed with sterilized distilled water supplemented with 500 mg/L cefotaxime and cultured on the solidified germination medium supplemented with 300 mg/L cefotaxime, 50 mg/L timentin and 150 mg/L kanamycin. Small shoots (1–2 cm height), appearing 40–60 days after transformation, were transferred to the rooting medium. Healthy looking plants (two to five true leaves, 2–5 cm in height, and a 2–15 cm tap root) were then moved to pots containing a vermiculite-perlite mixture (1:1 by volume) and further grown under greenhouse conditions. Regenerated plants were screened for the *Cas9* transgene by PCR using primers specified in **Supplementary Table 1**.

### Identification and characterization of Cas9-induced targeted mutations

Genomic DNA was extracted from leaves of WT and transgenic papaya plants following a CTAB-based method (Narzary et al., 2015). The *eIF4E* and *eIF(iso)4E* genomic regions spanning the Cas9 target sites were amplified by PCR using primers listed in **Supplementary Table 1**. Large amplicons spanning both the gRNA1 and gRNA2 target sites were separated on 2% agarose gels to detect deletions between gRNA1 and gRNA2, while, the short amplicons (spanning either gRNA1 or gRNA2) were subjected to heteroduplex analysis by native polyacrylamide gel electrophoresis (PAGE) to reveal indel mutations (Zhu et al., 2014). Briefly, amplified DNA from the WT and regenerated/gene edited plants were mixed in 1:1 ratio and then denatured at 95°C for 10 min, followed by gradual cooling down to room temperature. Next, the mixtures were separated in 15% native polyacrylamide gels, and the heteroduplexes between mutant and WT DNA strands were detected as shifted DNA bands. The induced mutations were further characterized by pJET cloning and Sanger sequencing on the ABI3500XL system (Applied Biosystems). *eIF4E* and *eIF(iso)4E* mutant sequences were compared with the corresponding WT sequences by BioEdit 7.2.

### Virus challenge

The PRSV isolate used in this study was collected from field papaya plants showing severe symptoms of PRSV infection in Ha Nam province (Vietnam), and the presence of PRSV was confirmed by RT-PCR using PRSV capsid protein (CP)-specific primers (**Supplementary Table 1**). Two T0 (*eIF4E* and *eIF(iso)4E*) mutant lines were *in vitro* multiplied to obtain 2–3 plants per line, and 10-week-old plants grown under greenhouse conditions (**Supplementary Figure 2**) were subjected to the virus challenge experiment as described previously (Le et al., 2022). Briefly, the infectious sap extracted from PRSV-infected papaya leaves was gently rubbed onto primary leaves of tested plants which had been dusted with silicon carbide powder. Inoculated WT plants and mock inoculated plants were used as positive and negative controls, respectively. After infection, the excess carborundum and sap were removed from leaves by rinsing with distilled water. Inoculated plants were sheltered from direct sunlight for 48h and maintained in the growth-chamber conditions at 25°C (± 2°C) under 16-h light/8-h dark photoperiod. PRSV accumulation in systemic leaves of all tested plants was evaluated at 20-, 30- and 40-days post-inoculation (dpi) by double-antibody sandwich enzyme-linked immunosorbent assay (DAS-ELISA) using a commercially available kit (BIOREBA, Germany) following manufacturer’s guidelines. The absorbance of the reaction mixtures was measured at 405 nm by a microplate reader, and the mean OD value at 405 nm of two or three biological replicates was used to estimate the PRSV titers in each papaya line. Data were graphed by GraphPad Prism 7.04 software and subjected to one-way ANOVA test (P values < 0.05) followed by Dunnett’s test for multiple comparisons. Plant growth and PRSV symptoms were continuously monitored up to 4 months post-inoculation under greenhouse condition.

### Sequence analysis of local PRSV isolate

Total RNA was extracted from PRSV-infected leaves using TRIzol reagent (Invitrogen, USA) following the manufacturer’s instruction. First-strand cDNA synthesis was performed with the HiScript III 1st Strand cDNA Synthesis Kit (+gDNA wiper) (Vazyme, China). The synthesized cDNA was then used as a template to amplify the coat protein (*CP*) gene of Papaya ringspot virus (PRSV) by PCR using primers PRSV-CP_F and PRSV-CP_R (**Supplementary Table 1**). The PCR product was purified using the GeneJET Gel Extraction Kit (Thermo Fisher Scientific, USA), ligated into the pJET1.2 plasmid using the CloneJET PCR Cloning Kit (Thermo Fisher Scientific, USA), and introduced into *E. coli* G10 competent cells by the heat-shock method. Plasmids were isolated using the GeneJET Plasmid DNA Purification Kit (Thermo Fisher Scientific, USA) and subsequently used for Sanger sequencing.

### qRT-PCR

Total RNA was isolated from leaves of WT and mutant papaya plants at 40 days post-inoculation using TRIzol Reagent (Invitrogen, USA) as instructed by the manufacturer. After that, cDNA was synthesized with the HiScript III 1st Strand cDNA Synthesis Kit (+gDNA wiper) (Vazyme, China). To quantify the PRSV accumulation in papaya lines, mRNA levels of the coat protein (*CP*) gene were explored by real-time quantitative PCR (qPCR) using GoTaq^®^qPCR Master Mix (Promega) on a Rotor-Gene Q real-time system (Qiagen). Each sample was analyzed in triplicate. The *TBP2* gene encoding papaya tata binding protein 2 was used as an internal reference gene for normalization, and relative expression levels of the PRSV *CP* gene were determined by Ct values and calculated using the 2-△△Ct method (Livak and Schmittgen, 2001). Primer information is provided in **Supplementary Table 1**. Data were plotted using GraphPad Prism software version 7.04, and one-way ANOVA with Dunnett’s test was performed, comparing each group to the WT (wild-type) plant.

## Results and discussion

### gRNA design and vector construction

Although the plant eukaryotic translation initiation factor 4E is recognized as a major mediator of potyvirus tolerance, the contribution of eIF4E and its isoform, eIF(iso)4E, to potyvirus resistance vary among different plant species. Therefore, in this study, we individually targeted both the *eIF4E* and *eIF(iso)4E* genes for mutagenesis using CRISPR/Cas9 (**Figure 1A**). For each gene, a dual gRNAs-CRISPR/Cas9 construct was designed, with the Cas9 endonuclease driven by the CaMV35S promoter, and two gRNAs controlled by the Arabidopsis U6 promoter (**Figure 1B**). In the *eIF4E* gene, gRNA1 and gRNA2 were selected to guide Cas9 to cleave at the second and the third exons, respectively, while the Cas9 target sites of the *eIF(iso)4E* were located within exon 1 and exon 2. Ideally, double strand DNA breaks at both target sites would result in large deletions (358 bp and 1303 bp for *eIF4E* and *eIF(iso)4E*, respectively) (**Figure 1A**) which can be readily detected by agarose gel electrophoresis of PCR amplicons (Do et al., 2019).

### Transgenic papaya generation

Next, the CRISPR/Cas9 constructs pKSE401-eIF4E and pKSE401-eIF(iso)4E, targeting *eIF4E* and *eIF(iso)4E*, respectively (**Figure 1B**) were used to transform the local papaya cultivar Linhan by *Agrobacterium*-mediated gene transfer. For each construct, two experimental batches were conducted, and both yielded shoots capable of regeneration on selective medium. The pKSE401-eIF4E construct was used to transform approximately 1000 explants, resulting in 6 shoot-forming explants from about 130 explants that survived on media containing 150 mg/L kanamycin. Similarly, approximately 750 explants were transformed with the pKSE401-eIF(iso)4E construct, with around 60 explants surviving on medium supplemented with 150 mg/L kanamycin. Of these, 12 explants were capable of forming shoots and rooted when transferred to the rooting medium. Some regenerated plants struggled with root development, while others displayed altered morphology. Notably, two plants from each group exhibited a wild-type (WT) phenotype (**Supplementary Figure 2**) and were subject to subsequent analyses.

### Characterization of Cas9-induced mutations in the *eIF4E* and *eIF(iso)4E* genes in T0 transgenic papaya

**Figure 2:**
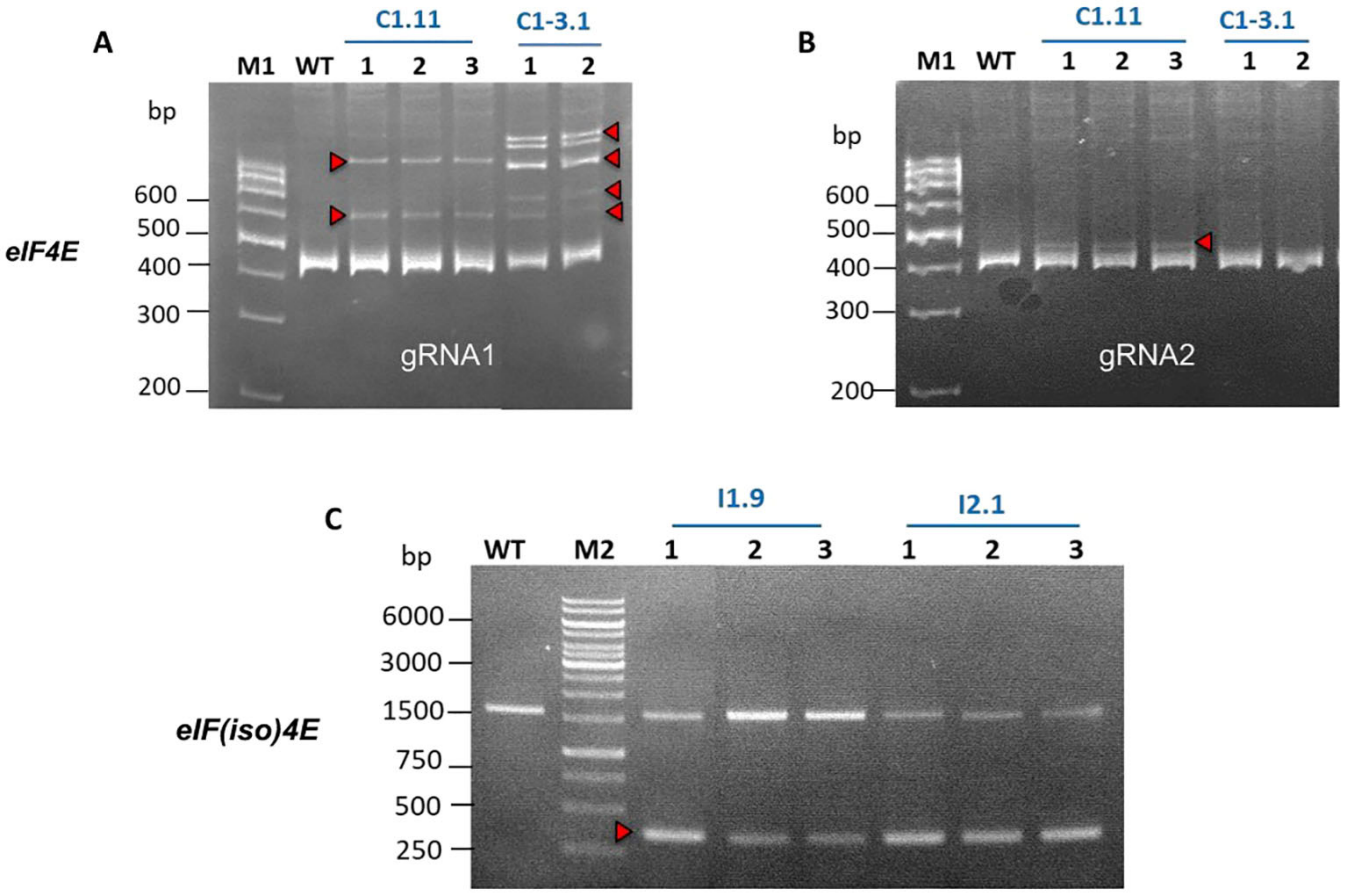
Characterization of CRISPR/Cas9-induced mutations in the *eIF4E* and *eIF(iso)4E* genes in transgenic papaya T0 lines. **(A, B)** Detection of indels in the target regions of *eIF4E*-gRNA1 and *eIF4E*-gRNA2 by heteroduplex analysis. **(C)** Identification of DNA lesions in the *eIF(iso)4E* gene by PCR. C1.11, C1-3.1: *eIF4E* mutant lines; I1.9, I2.1: *eIF(iso)4E* mutant lines; WT: wild-type plant; M1: 100 bp DNA ladder (Thermo Scientific); M2: 1 kb DNA ladder (Thermo Scientific). The red arrows indicate the presence of CRISPR/Cas9-mediated mutations. The vegetatively propagated clones (different plants) are numbered 1-3.

First, Cas9-induced mutations in the target genes were assessed by 2% agarose gel electrophoresis of PCR-generated amplicons spanning both gRNA target sites (**Figure 1A**). Low molecular weight DNA bands indicating the expected large deletions were observed only in the *eIF(iso)4E*-edited papaya lines I1.9 and I2.1 (**Figure 2C**, red arrow). In contrast, no large deletions were detected in the *eIF4E*-edited lines C1.11 and C1-3.1 (**Supplementary Figure 3**). Therefore, heteroduplex analysis was conducted to detect indel mutations around the gRNA1 (**Figure 2A**) and gRNA2 (**Figure 2B**) target regions in the *eIF4E* gene. Shifted DNA bands on PAGE gels, compared with the WT DNA band, confirmed that line C1.11 harbored indels at both target sites in *eIF4E*, whereas C1-3.1 contained a mutation only at the gRNA1 target site (**Figures 2A,B**). From these results, we conclude that our gene editing reagents were specific and active; however, the absence of large deletions in eIF4E may be attributed to the lower activity of gRNA2.

**Figure 3:**
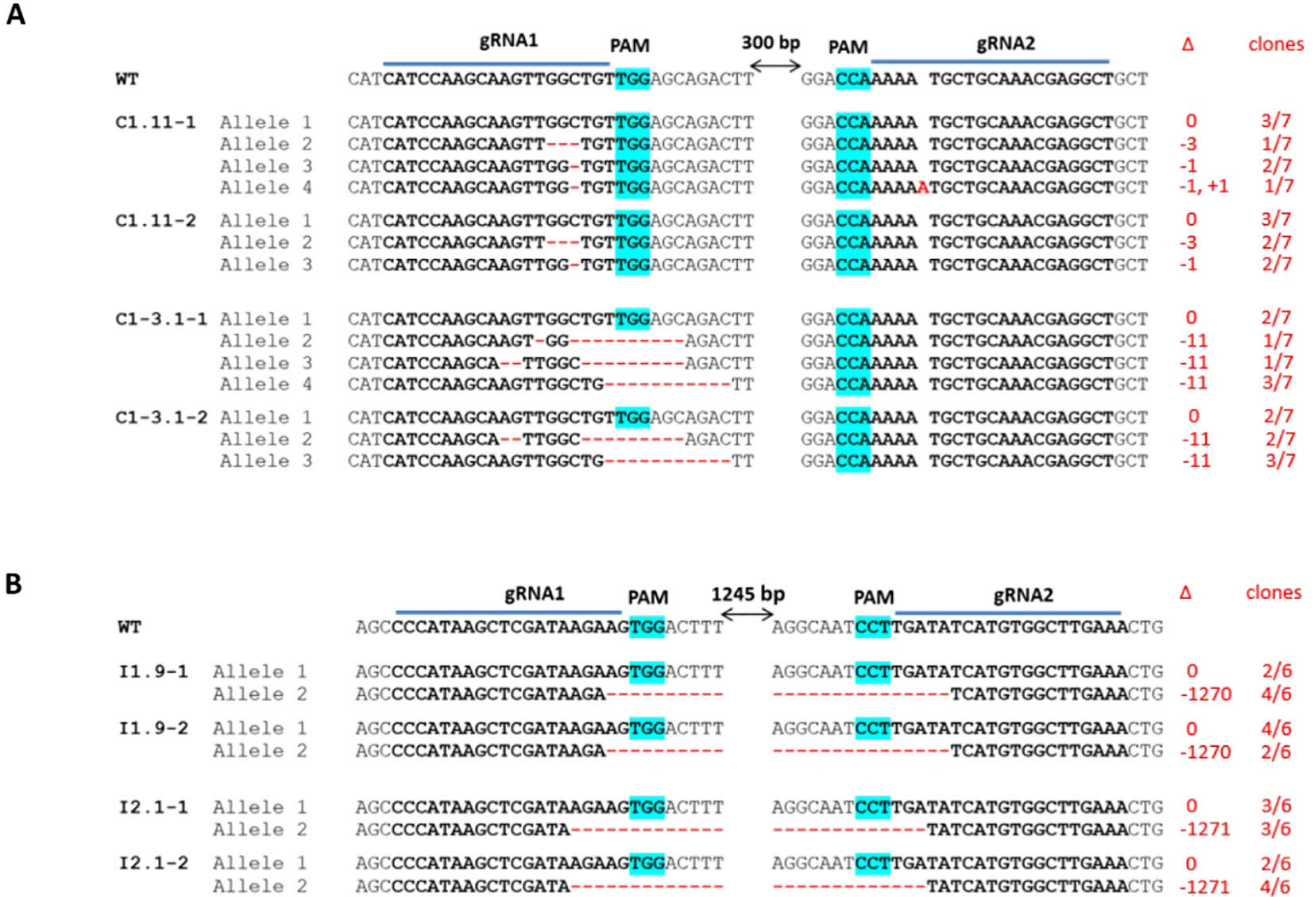
Sequence analysis of the CRISPR/Cas9 target sites in the *eIF4E-* and *eIF(iso)4E*-edited T0 transgenic papaya lines. **(A)** Sequence alignment of CRISPR/Cas-induced mutations at the gRNA1 target site in the *eIF4E-*edited lines C1.11 and C1-3.1 **(B)** Sequence alignment of CRISPR/Cas-induced mutations between the gRNA1 (left panel) and gRNA2 (right panel) target sites in the *eIF(iso)4E*-edited lines I1.9 and I2.1. WT: wild-type plant. gRNA target sequences are shown in bold, and PAM motifs are highlighted in magenta. Red letters indicate inserted nucleotides, and red dashed lines indicate deletions. ∆ represents the length of indels (nt) detected at the CRISPR/Cas9 target sites, the symbols used are ‘0’ for no change, ‘-’ for a deletion, and ‘+’ for an insertion. Vegetative clones are labeled as #-1 and #-2. The allele frequency, shown on the right, is calculated based on the number of clones sequenced from each plant.

CRISPR/Cas9-induced mutations in each target gene were subsequently characterized by Sanger sequencing using two independently propagated vegetative clones of the selected T0 lines, labeled as #-1 and #-2 (**Figure 3**). Consistent with the gel electrophoresis results, the sequencing data revealed heterozygous mutations involving -1270 bp and -1271 bp deletions in *eIF(iso)4E* in the I1.9 and I2.1 lines, respectively (**Figure 3B**). Moreover, chimeric mutations were identified in the *eIF4E* gene in both the C1.11 and C1-3.1 lines. Besides the WT allele, line C1.11 carried two single-mutation alleles of -3 bp and -1 bp at the gRNA1 target site and a double-mutation allele (-1 bp and +1 bp at gRNA 1 and gRNA2 sites, respectively) (**Figure 3A**), while line C1-3.1 harbored three types of 11 bp deletions at the gRNA1 cut site in *eIF4E* (**Figure 3A**). No sequence variation was observed between the vegetative clones, which suggests that Cas9-induced mutations are stable in the vegetatively propagated progeny.

### Assessing PRSV resistance of the *eIF4E* and *eIF(iso)4E*-edited T0 papaya lines

To examine the impact of CRISPR/Cas9-induced mutations on PRSV resistance in papaya, the *eIF4E-* and *eIF(iso)4E-*edited T0 lines (**Figure 3**; **Supplementary Figure 2**) were challenged with a local PRSV isolate, which shares very high sequence similarity with previously described PRSV isolates from Asia-Pacific countries (**Supplementary Figures 4**, **5**).

**Figure 4:**
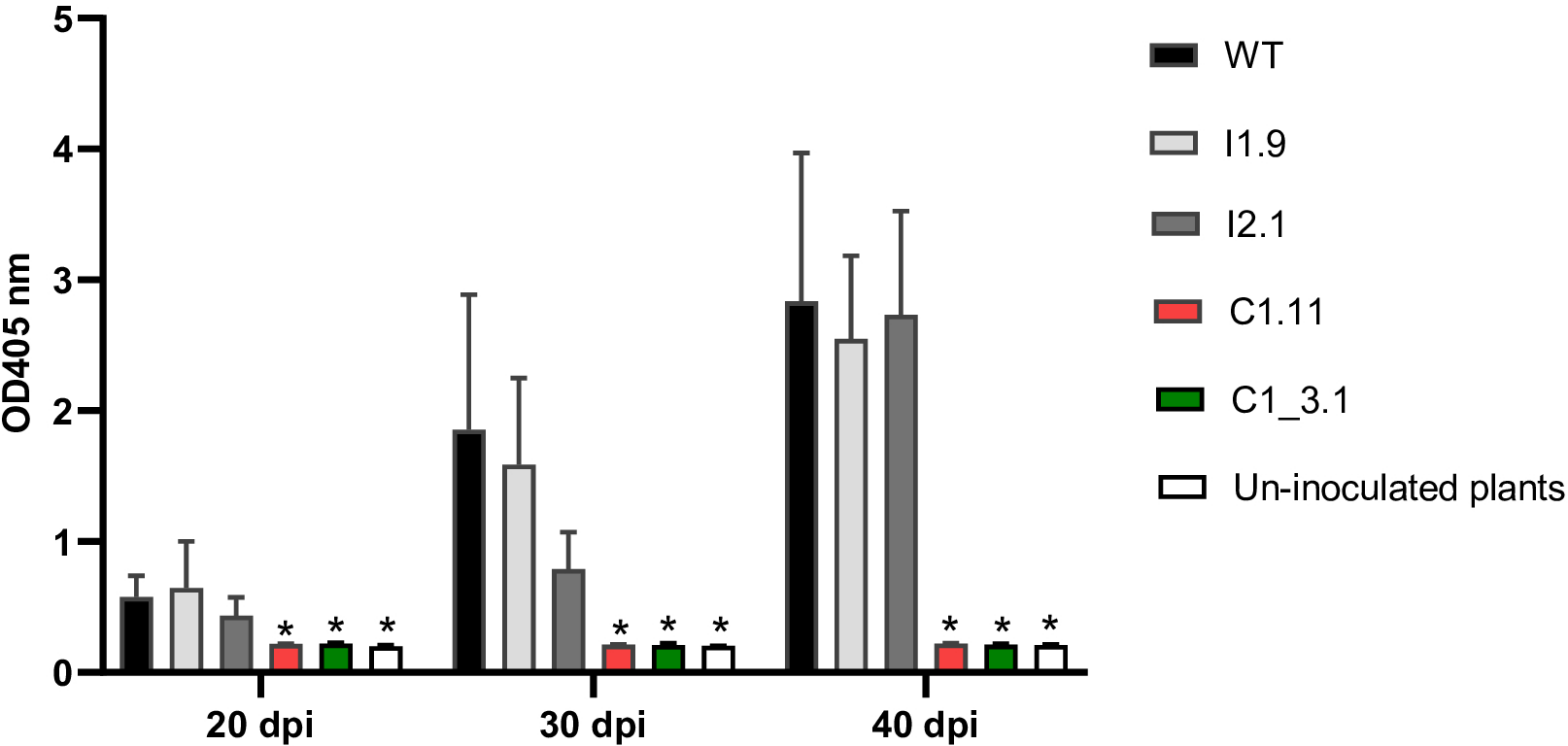
Analysis of PRSV accumulation in T0 papaya plants by Double Antibody Sandwich ELISA (DAS-ELISA). PRSV accumulation was quantified at 20-, 30-, and 40-days post-inoculation (dpi) in the leaves of tested T0 lines. WT: wild-type plants; I1.9 and I2.1: *eIF(iso)4E* mutant lines; C1.11 and C1-3.1: *eIF4E* mutant lines. Mean A405 values and standard errors were calculated from 2 or 3 independent plants per line. Asterisk (*) indicates significant differences (P < 0.05) from the wild-type control, as determined by one-way ANOVA test followed by Dunnett’s test for multiple comparisons.

**Figure 5:**
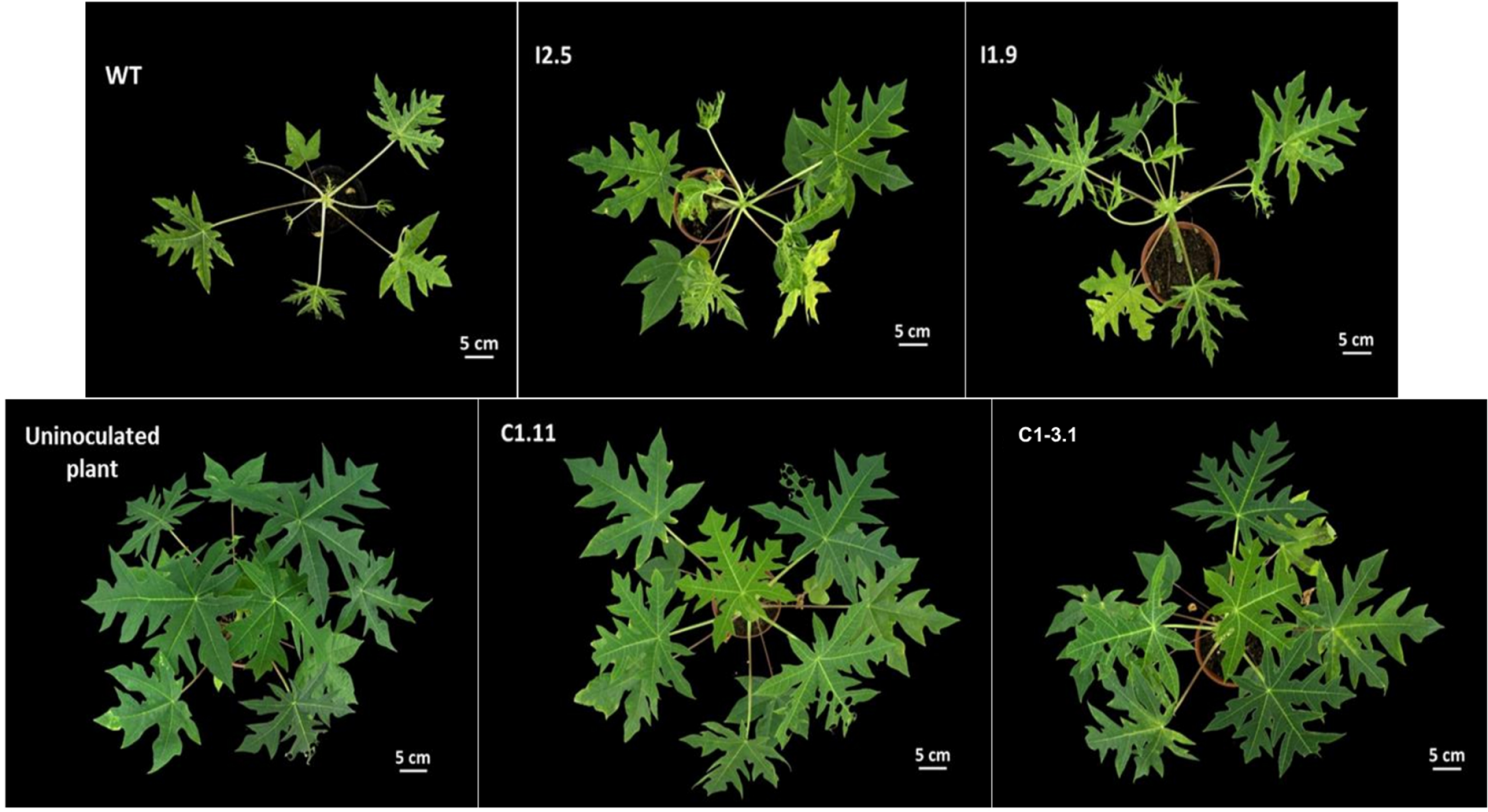
Assessing PRSV susceptibility of the gene edited T0 lines. Plants were infected with PRSV and photographed at 40 days post inoculation (dpi). C1.11, C1-3.1: *eIF4E* mutant lines; I1.9, I2.1: *eIF(iso)4E* mutant lines; WT: wild-type plant.

**Figure 6:**
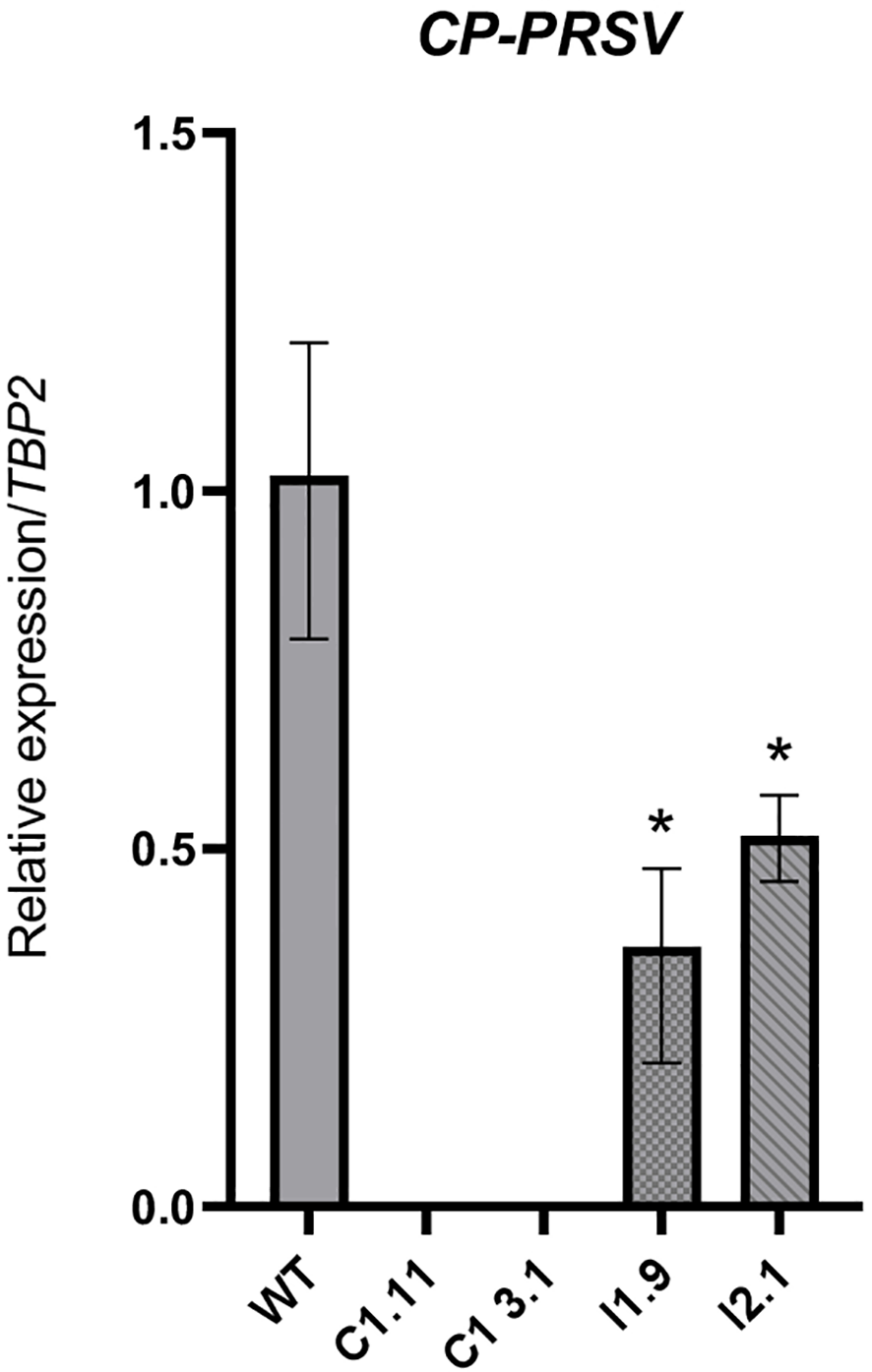
Quantitative real-time PCR analysis of PRSV accumulation in infected papaya leaves. The PRSV accumulation was quantified at 40 days post-inoculation. C1.11 and C1-3.1: *eIF4E* mutant lines; I1.9 and I2.1: *eIF(iso)4E* mutant lines; WT: wild-type plant. Asterisk (*) indicates significant differences (P < 0.05) from the wild-type control, as determined by one-way ANOVA test followed by Dunnett’s test for multiple comparisons.

20 days after inoculation, low-level PRSV titers were detected in the systemic leaves of WT plants as well as in all tested plants of both *eIF(iso)4E*-edited lines (I1.9, I2.1). The virus accumulation in these papaya lines remarkably increased, nearly 2 and 3 times, at 30- and 40-days post inoculation (dpi), respectively, indicating their susceptibility to PRSV (**Figure 4**, **Supplementary Table 3**). All virus-infected plants developed typical symptoms of PRSV disease such as stunted growth, leaf distortion and reduction, vein clearing and mosaic (**Figure 5**). Importantly, no PRSV infection was detected in the *eIF4E*-edited lines (C1.11, C1-3.1) at any time points tested by ELISA (**Figure 4**, **Supplementary Table 3**). To assess viral RNA levels in PRSV-infected plants, we also performed qRT-PCR at 40 dpi. In agreement with the phenotypic and colorimetric data (**Figures 4**, **5**), WT and *eIF(iso)4E*-edited plants showed very high virus titers. In contrast, *eIF4E* mutants contained no detectable PRSV RNA (**Figure 6**). At the final monitoring time point (4 months under net-house conditions), all vegetatively propagated plants of C1.11 and C1-3.1 lines grew normally and showed no symptoms of PRSV disease (**Supplementary Figure 6**). All in all, our data demonstrate that frameshift mutations in the *eIF4E* gene confer highly efficient PRSV resistance to papaya plants.

eIF-mediated resistance to potyviruses has been reported in many plants (Julio et al., 2014; Chandrasekaran et al., 2016; Wang et al., 2013; Li et al., 2023, Li et al., 2024; Liu et al., 2024; Suzuki et al., 2025). The molecular and cellular mechanisms of this resistance are based on the mode of potyvirus infection, wherein viral genome-linked protein (VPg) must interact with eukaryotic translation initiation factors (eIFs) such as eIF4E or eIF(iso)4E to initiate the translation of viral genomic RNA. Because this interaction is essential for virus replication and spread, the down-regulation or mutagenesis of genes encoding these host factors often induces recessive resistance, categorized as a “loss-of-susceptibility” (Shopan et al., 2020). Our results indicate the major involvement of eIF4E, rather than eIF(iso)4E, in the infection of papaya by PRSV. This finding is consistent with a previous report in which two amino acid changes (L227H and S230G) in the eIF4E of the resistant highland wild papaya, *Vasconcellea cauliflora*, were found to be associated with PRSV resistance (Harini et al., 2019). Interestingly, our data also revealed that chimeric editing of *eIF4E* was sufficient to confer PRSV resistance in T0 lines, as demonstrated by ELISA and qRT-PCR assays. This suggests that a single mutated allele (heterozygous) might reduce the total amount of functional susceptibility factor below the threshold required for efficient virus replication. Alternatively, mosaic/chimeric mutations may lead to dominant negative versions of eIF4E proteins that are unable to bind VPg. Such mutant proteins could interfere with the function of the wild-type eIF4E (produced from the susceptible allele) by forming non-functional multimers with the eIF4F complex, effectively “poisoning” the translation machinery. Similar phenomena, where chimeric mutations confer host protection, have been reported previously; for example, grapevines carrying chimeric mutant *edr1* alleles displayed enhanced resistance to powdery mildew without developmental defects (Yu et al., 2024). Notably, heterozygous mutations in our *eIF(iso)4E* lines (I1.9 and I2.1) retained high virus susceptibility. However, the qPCR data indicated significantly lower viral accumulation in these lines, suggesting a potential interplay between the two isoforms in papaya, consistent with observations in other plant species (Udagawa et al., 2021; Suzuki et al., 2025). Due to limitations in utilizing T0 chimeric mutant lines, developing additional lines with homozygous or biallelic mutations that are transgene-free will be necessary to fully elucidate the individual roles of eIF4E and eIF(iso)4E in the PRSV life cycle. Given that eIF4E-mediated resistance can be highly strain-specific (Udagawa et al., 2021), further tests with other PRSV isolates from Vietnam and around the world are necessary to assess the potential of these CRISPR/Cas9-induced mutations for enhancing durable resistance in papaya to diverse global PRSV phylogroups.

In conclusion, we targeted the papaya *eIF4E* and *eIF(iso)4E* genes for individual mutagenesis using the CRISPR/Cas9 technology. All plants with edited *eIF4E* genes - but none with edited *eIF(iso)4E* genes - were protected against PRSV, confirming the dominant role of eIF4E in papaya PRSV infection. However, further studies are required to assess the impact of homozygous loss-of-function alleles on PRSV resistance, as well as the effects of these eIF4E mutations across the entire life cycle and under varied stress conditions. Nevertheless, these finding provide a novel strategy to enhance papaya resistance to PRSV and inform future precision breeding efforts, including base and prime editing, to develop improved local cultivars.

## Data Availability

The datasets generated during and/or analysed during the current study are available from the corresponding author on reasonable request.
