# Supplementary material for: Enhancing papaya resistance to ringspot virus through CRISPR/Cas9-mediated gene editing of eIF4E
Source: Front Plant Sci. 2026 May 19;17:1799408. doi: 10.3389/fpls.2026.1799408 (PMC13226507; doi:10.3389/fpls.2026.1799408)
Supplement: Supplementary file 1 [file DataSheet1.docx]

**Supplementary Information**

**Table S1.** List of primers used in this study

The targeted sequences are in red and the *Bsa*I restriction site is underlined

**Table S2.** Composition of different media used in this study

**Table S3.** Quantification of PSRV infection over time in the *eIF4E* and *eIF(iso)4E* mutant lines

dpi: days post-inoculation

**Figure S1.** Steps of *Agrobacterium*-mediated transformation and transgenic papaya plant regeneration. (A) Papaya immature seeds. (B) Papaya zygotic embryos excised from seeds. (C) Callus induction from zygotic embryos. (D) Somatic embryo formation. (E) Inoculation and co-cultivation. (F) Selection. (G) Shoot induction. (H) Transgenic plant regeneration.

**Figure S2.** Phenotype of the papaya T0 lines photographed before PRSV inoculation. C1.11, CR1-3.1: *eIF4E* mutant lines; I1.9, I2.1: *eIF(iso)4E* mutant lines; WT: wild-type plant

**Figure S3.** Analysis of DNA lesion in the *eIF4E* gene by PCR**.** PCR products were separated in a 2% agarose gel. IC1.11, C1-3.1: *eIF4E* mutant lines; WT: wild-type plant; M1: 100 bp DNA ladder (Thermo Scientific). The vegetatively propagated clones are numbered 1 to 3.

PRSV-VNP-03 GGTCTGAATGAAAAGCTAAAAGAGAAAGAAAAACAGAAAGAGAAAGAAAAAGAAAAAGAT

clone2 GGTCTGAATGAAAAGCTGAAAGAAAAGGGAAAACAAAAAGATAAAGAAAAAGAAAAAGAT

clone1 GGTCTGAATGAAAAGCTGAAAGAAAAGGAAAAACAAAAAGATAAAGAAAAAGAAAAAGAT

clone3 GGTCTGAATGAAAAGCTGAAAGAAAAGGAAAAACAAAAAGATAAAGAAAAAGAAAAAGAT

***************** ***** ** * ****** ***** ******************

PRSV-VNP-03 AAACAAAAAGATAAAGATAATGATGGAGCTAGTGACGGAAATGATGTGTCAACTAGCACA

clone2 AAACAAAAAGAAAAAGATAATGATGGAGCTAGTGACGGAAAGGATGTGTCAACTAGCACA

clone1 AAACAAAAAGAAAAAGATAATGATGGAGCTAGTGACGGAAAGGATGTGTCAACTAGCACA

clone3 AAACAAAAAGAAAAAGATAATGATGGAGCTAGTGACGGAAAGGATGTGTCAACTAGCACA

*********** ***************************** ******************

PRSV-VNP-03 AAAACTGGAGAGAGAGATAGAGATGTCAACGCCGGAACTAGTGGAACTTTCACTGTTCCA

clone2 AAAACTGGAGAGAGAGATAGAGATGTCAACGCCGGAACTAGTGGAACTTTCACAGTTCCA

clone1 AAAACTGGAGAGAGAGATAGAGATGTCAACGCCGGAACTAGTGGAACTTTCACAGTTCCA

clone3 AAAACTGGAGAGAGAGATAGAGATGTCAACGCCGGAACTAGTGGAACTTTCACAGTTCCA

***************************************************** ******

PRSV-VNP-03 AGGATAAAGTCTTTTACTGACAAGATGATTTTACCAAGAATTAAGGGAAAGTCTGTTCTT

clone2 AGGATAAAGTCTTTTACTGACAAGATGATTTTACCAAAAATTAAGGGAAAGTCTGTCCTT

clone1 AGGATAAAGTCTTTTACTGACAAGATGATTTTACCAAAAATTAAGGGAAAGTCTGTCCTT

clone3 AGGATAAAGTCTTTTACTGACAAGATGATTTTACCAAAAATTAAGGGAAAGTCTGTCCTT

************************************* ****************** ***

PRSV-VNP-03 AATTTAAATCATCTTCTTCAGTATAATCCGCAACAAATTGACATCTCAAACACTCGTGCC

clone2 AATTTAAATCATCTTCTTCAGTATAATCCGCAACAAATTGACATCTCAAACACTCGTGCC

clone1 AATTTAAATCATCTTCTTCAGTATAATCCGCAACAAATTGACATCTCAAACACTCGTGCC

clone3 AATTTAAATCATCTTCTTCAGTATAATCCGCAACAAATTGACATCTCAAACACTCGTGCC

************************************************************

PRSV-VNP-03 ACTCAATCTCAATTTGAAAAGTGGTATGAGGGAGTGAGGAATGATTATGGTCTTAGTGAT

clone2 ACTCAATCTCAATTTGAAAAGTGGTATGAGGGAGTGAGGAATGATTATGGTCTTAGTGAT

clone1 ACTCAATCTCAATTTGAAAAGTGGTATGAGGGAGTGAGGAATGATTATGATCTTAGTGAT

clone3 ACTCAATCTCAATTTGAAAAGTGGTATGAGGGAGTGAGGAATGATTATGGTCTTAGTGAT

************************************************* **********

PRSV-VNP-03 AACGAAATGCAAGTGATGTTAAATGGTTTGATGGTTTGGTGTATCGAAAATGGTACATCT

clone2 AACGAAATGCAAGTGATGTTAAATGGTTTGATGGTTTGGTGTATCGAAAATGGTACATCT

clone1 AACGAAATGCAAGTGATGTTAAATGGTTTGATGGTTTGGTGTATCGAAAATGGTACATCT

clone3 AACGAAATGCAAGTGATGTTAAATGGTTTGATGGTTTGGTGTATCGAAAATGGTACATCT

************************************************************

PRSV-VNP-03 CCAGACATATCTGGTGTTTGGGTAATGATGGATGGGGAAACTCAGGTCGATTATCCCATC

clone2 CCAGACATATCTGGTGTTTGGGTAATGATGGATGGGGAAATTCAGGTCGATTATCCCATT

clone1 CCAGACATATCTGGTGTTTGGGTAATGATGGATGGGGAAATTCAGGTCGATTATCCCATT

clone3 CCAGACATATCTGGTGTTTGGGTAATGATGGATGGGGAAATTCAGGTCGATTATCCCATT

**************************************** ******************

PRSV-VNP-03 AAACCTTTAATTGAACATGCAACTCCTTCATTCAGGCAAATCATGGCTCACTTCAGTAAC

clone2 AAACCTTTAATTGAACATGCAACTCCTTCATTCAGGCAAATCATGGCTCACTTCAGTAAC

clone1 AAACCTTTAATTGAACATGCAACTCCTTCATTCAGGCAAATCATGGCTCACTTCAGTAAC

clone3 AAACCTTTAATTGAACATGCAACTCCTTCATTCAGGCAAATCATGGCTCACTTCAGTAAC

************************************************************

PRSV-VNP-03 GCGGCAGAGGCATATATCGCGAAGAGAAATGCAACTGAGAGGTACATGCCGCGGTATGGA

clone2 GCGGCAGAGGCATACATCGCGAAGAGAAATGCAACTGAGAGGTACATGCCGCGGTATGGA

clone1 GCGGCAGAGGCATACATCGCGAAGAGAAATGCAACTGAGAGGTACATGCCGCGGTATGGA

clone3 GCGGCAGAGGCATACATCGCGAAGAGAAATGCAACTGAGAGGTACATGCCGCGGTATGGA

************** *********************************************

PRSV-VNP-03 ATTAAAAGGAATTTGACTGACATTAGTCTCGCTCGATATGCTTTCGATTTCTATGAGGTG

clone2 ATTAAAAGGAATTTGACTGACATTAGTCTCGCTCGATATGCTTTCGATTTTTATGAGGTG

clone1 ATTAAAAGGAATTTGACTGACATTAGTCTCGCTCGATATGCTTTCGATTTTTATGAGGTG

clone3 ATTAAAAGGAATTTGACTGACATTAGTCTCGCTCGATATGCTTTCGATTTTTATGAGGTG

************************************************** *********

PRSV-VNP-03 AATTCGAAAACACCTGATAGGGCTCGTGAAGCTCATATGCAGATGAAAGCTGCAGCTCTA

clone2 AATTCGAAAACACCTGATAGGGCTCGTGAAGCTCATATGCAGATGAAAGCTGCAGCTTTG

clone1 AATTCGAAAACACCTGATAGGGCTCGTGAAGCTCATATGCAGATGAAAGCTGCAGCTTTG

clone3 AATTCGAAAACACCTGATAGGGCTCGTGAAGCTCATATGCAGATGAAAGCTGCAGCTTTG

********************************************************* *

PRSV-VNP-03 CGTAATGCTAGTCGCAGAATGTTCGGAATGGACGGCAGTGTCAGTAACAAGGAAGAAAAC

clone2 CGTAATGCTAGTCGCAGAATGTTCGGAATGGACGGCAGTGTCAGTAACAAGGAAGAAAAC

clone1 CGTAATGCTAGTCGCAGAATGTTCGGAATGGACGGCAGTGTCAGTAACAAGGAAGAAAAC

clone3 CGTAATGCTAGTCGCAGAATGTTCGGAATGGACGGCAGTGTCAGTAACAAGGAAGAAAAC

************************************************************

PRSV-VNP-03 ACGGAGAGACACACAGTGGAAGATGTCAACAGAGACATGCACTCTCTCCTGG

clone2 ACGGAGAGACACACAGTGGAAGATGTCAACAGAGACATGCACTCTCTCCTGG

clone1 ACGGAGAGACACACAGTGGAAGATGTCAACAGAGACATGCACTCTCTCCTGG

clone3 ACGGAGAGACACACAGTGGAAGATGTCAACAGAGACATGCACTCTCTCCTGG

****************************************************

**Figure S4.** Alignment of the coat protein–specific sequences of the PRSV isolate used in this study with the GenBank accession AF506888.1 (PRSV-VNP-03) by CLUSTAL multiple sequence alignment MUSCLE (3.8).

**Figure S5.** Phylogenetic tree of PRSV CP gene sequences constructed in MEGA6 using the neighbour-joining method. The phylogenetic tree was based on partial coat-protein sequences of 24 PRSV isolates from Vietnam, China, Thailand, the Philippines, Taiwan, Japan, India, Colombia, Hawaii, France, the USA, Ecuador, and Australia. Papaya ringspot virus type W (PRSV-W) and potato virus Y (PVY) were included as outgroups. The PRSV strain isolated for the virus-challenge experiment is indicated by the red rectangle.

**Figure S6.** Phenotype of WT and papaya T0 lines photographed 4 months after PRSV inoculation.
